# Effects of health and safety problem recognition on small business facility investment

**DOI:** 10.1186/2052-4374-25-26

**Published:** 2013-10-23

**Authors:** Jisu Park, Harin Jeong, Sujin Hong, Jong-Tae Park, Dae-Sung Kim, Jongseo Kim, Hae-Joon Kim

**Affiliations:** 1 Department of Occupational and Environmental Medicine, College of Medicine, Korea University, 123, Jeokgeum-ro, Danwon-gu, Ansan-si, Gyeonggi-do 425-707, Republic of Korea; 2 Department of Preventive Medicine, College of Medicine, Korea University, 73, Inchon-ro, Seongbuk-gu, Seoul 136-705, Republic of Korea

**Keywords:** Small business, Accident prevention, Occupational health, Facility investment

## Abstract

**Objectives:**

This study involved a survey of the facility investment experiences, which was designed to recognize the importance of health and safety problems, and industrial accident prevention. Ultimately, we hope that small scale industries will create effective industrial accident prevention programs and facility investments.

**Methods:**

An individual survey of businesses’ present physical conditions, recognition of the importance of the health and safety problems, and facility investment experiences for preventing industrial accidents was conducted. The survey involved 1,145 business operators or management workers in small business places with fewer than 50 workers in six industrial complexes.

**Results:**

Regarding the importance of occupational health and safety problems (OHS), 54.1% said it was “very important”. Received technical and financial support, and industrial accidents that occurred during the past three years were recognized as highly important for OHS. In an investigation regarding facility investment experiences for industrial accident prevention, the largest factors were business size, greater numbers of industrial accidents, greater technical and financial support received, and greater recognition of the importance of the OHS. The related variables that decided facility investment for industry accident prevention in a logistic regression analysis were the experiences of business facilities where industrial accidents occurred during the past three years, received technical and financial support, and recognition of the OHS. Those considered very important were shown to be highly significant.

**Conclusions:**

Recognition of health and safety issues was higher when small businesses had experienced industrial accidents or received financial support. The investment in industrial accidents was greater when health and safety issues were recognized as important. Therefore, the goal of small business health and safety projects is to prioritize health and safety issues in terms of business management and recognition of importance. Therefore, currently various support projects are being conducted. However, there are issues regarding the limitations of the target businesses and inadequacies in maintenance and follow-up. Overall, it is necessary to provide various incentives for onsite participation that can lead to increased recognition of health and safety issues and practical investments, while perfecting maintenance and follow up measures by thoroughly revising existing operating systems.

## Introduction

The definition of a small business may vary depending on the purpose, but it is commonly categorized based on the size of business capital investment and the number of workers. The term small business has been defined by the International Labour Organization (ILO) in 1986 as a business with fewer than 50 employees [1]. The Korea Occupational Safety and Health Act is also applied on the basis of this generally recognized definition.

The current status of domestic small businesses with fewer than 50 employees can be examined through the 2013 National Business Survey reported by Statistics Korea. According to this survey, the number of businesses that satisfy the definition of a small business, i.e., having fewer than 50 employees, was 6,855,692 or 98.7%, while the number of employees working in those businesses amounted to 23,434,782 or 64.7% [2].

Survey results in other countries are not significantly different from this domestic survey. In case of Japan, the 2006 statistics revealed that small businesses with fewer than 50 employees accounted for a very high percentage with 96.7% of all businesses and 60.6% of all workers [3]. In the United States, the classification criterion for a small business is fewer than 100 employees, rather than fewer than 50 employees as it is in Korea. According to the 2008 statistics, 89.2% of these U.S. businesses employed fewer than 20 employees while a majority of businesses (98%) had fewer than 100 workers. The number of employees working in businesses with fewer than 100 employees was 34.8% [4].

The number of domestic industrial accidents somewhat decreased from 97,821 cases in 2009 to 93,292 cases in 2011, but the percentage of injuries for small businesses increased from 79.6% in 2009 to 82.4% in 2011 [5]. This shows that small businesses are vulnerable to industrial accidents and that focus must be given to the development of safety and health activities for small businesses when establishing occupational health policies.

In order to prevent industrial accidents in small businesses with poor management and working conditions, the key point is that the employer and the employees in each business recognize the importance of safety and health issues and secure practical investment in facilities for industrial accident prevention. Previous domestic research on business health management has dealt with various aspects for improving the health management system and the overall fact finding on the business health management in small and medium-sized businesses thus far [6-16]. However, there is limited ongoing research and discussion on how to motivate facility investment for industrial accident prevention in each small business. Such research is necessary to focus on facility investment to prevent industrial accidents for future small businesses. Against this backdrop, this study aims to implement an effective industrial accident prevention program and secure investment in small business facilities. To achieve this, we garnered views on the effects of perceptions regarding safety and health issues on facility investment by conducting a survey among small businesses with fewer than 50 employees.

## Materials and methods

### Research subjects and research method

Six regions were selected for this study’s population–Gyeonggi (Banwol Sihwa Industrial Complex), Seoul (Guro Digital Industrial Complex), Gyeongnam (Gimhae Industrial Complex), Daegu (Seongseo Industrial Complex), Gwangju (Hanam Industrial Complex), and Chungnam (Asan Industrial Complex). These regions are representative of all small businesses after taking into consideration the regional distribution among industrial complexes with more than 1,000 small businesses that have fewer than 50 employees or with more than 20,000 employees. The samples were distributed for each region based on the business database for this population, and businesses were randomly selected using a stratified sampling method with type of business and business size as the stratifying variables. Based on the businesses selected in this process, interviews were conducted for two months from October to November 2010 using a structured questionnaire administered by professional interviewers who had prior contact. The interviewees were employers or the managerial-level employees who worked in manufacturing and non-manufacturing industries. The total number of interviewees were 1,253 (maximum permissible error of ±2.77% at a 95% confidence level). Among these, 95 cases of non-manufacturing and 13 cases of no response, which were treated as missing values, were excluded from the analysis due to their insignificance. Thus, 1,145 workers of manufacturing units were included in the final analysis.

The survey items included general information regarding the business (such as industry, location, number of employees, type of employment, type of work, and whether hazardous agents were handled), the level of awareness regarding safety and health issues, incidences of industrial accidents over the past three years, and facility investment for industrial accident prevention. Regarding the level of awareness on safety and health issues, a 5-point scale was used for the question, “Do you think safety and health issues of workers are important”? The response, “it is very important” was regarded as the high interest in safety and health issues. For the analysis, this was categorized as one category of response, and the rest of the responses were categorized as others. We also surveyed the principal and impeding factors for resolving safety and health issues, and whether a business had benefited from the technological and financial support from the Ministry of Employment and Labor Safety.

### Analysis method

A frequency analysis was performed to identify the characteristics of each business, while a *χ*^2^ test was performed to identify the percentage of awareness regarding the importance of safety and health issues, facility investment experience for industrial accident prevention, technology, and financial support for each factor. The work-related characteristics of a business as well as the safety and health issues characteristics were independent variables. Simple and multiple logistic regression analyses were performed with the experience of investing in industrial accident prevention facilities in the past three years as the dependent variable. Collected data were statistically analyzed using SPSS 18.0 for Windows and the significance level was under 0.05.

## Results

### General characteristics of the research subjects

Regarding the general characteristics of the businesses, the categories by industry were as follows: machine and metal (*n* = 488, 42.7%), light industries (*n* = 284, 24.9%), electrical and electronics (199, 17.5%), and petrochemical (*n* = 170, 14.9%). By size, businesses with 10–29 employees were the greatest (*n* = 375; 33.0%), followed by 5–9 employees (*n* = 328; 28.8%). By employment type, the number of businesses with only full-time employment was 981 (85.8%), while those with part-time employment were 162 (14.2%). By work type, the number of businesses with normal working hours without shift changes accounted for the most with 87.1% (Table 1).

**Table 1:** General characteristics of study subjects

**Characteristics**	**Frequency (%)**
Region (n = 1,145)	
Gyeonggi	305(26.6)
Seoul	170(14.8)
Gyeongnam	254(22.2)
Daegu	182(15.9)
Gwangju	144(12.6)
Chungnam	90(7.9)
Type of industry (n = 1,145)	
Light*	284(24.9)
Petrochemical	170(14.9)
Machine and metal	488(42.7)
Electrical and electronics	199(17.5)
Company size (no. of employees) (n = 1,141)	
≦4	285(24.9)
5-9	330(28.8)
10-29	376(32.8)
30-49	150(13.1)
Type of employment (n = 1,143)	
Regular only	981(85.8)
With irregular	162(14.2)
Shift work (n = 1,143)	
No	995(87.1)
Yes	148(12.9)

*Light: Manufacture of Food Products, Manufacture of Beverages, Manufacture of Luggage and Footwear etc.

### Level of awareness regarding safety and health issues

In the survey asking about the awareness of safety and health issues in the business, the responses “not important” or “not very important” were found to be very few with 5.3%. Therefore, taking into account that such responses are rarely encountered, the response “very important” was regarded as demonstrating particularly high interest in safety and health issues. In the analysis, we categorized responses into “very important” and other responses. As a result, the response “very important” accounted for 54.1%. In terms of region, this response was the highest in Gyeongnam and Chungnam, while petrochemical showed the highest industry response. The percentage of the response “very important” increased as the size of the businesses grew, but it was not statistically significant. In addition, a significant difference was found among the businesses receiving technical and financial support for safety and health management and those that did not. Those who did receive support and who answered “very important” were 69.1%, whereas those who did not receive support and answered “very important” were 49.5%. In addition, those businesses with industrial accidents in the past three years were somewhat more aware of the safety and health issues at work as compared to businesses that had not experienced an industrial accident (Table 2).

**Table 2:** Perception of importance of OHS^*^

	**Very important**		**Others**		**p-value**^**†**^
	**N**	**%**	**N**	**%**	
Total	616	54.0	525	46.0	
Region (n = 1,141)					<0.001
Gyeonggi	178	58.6	126	41.4	
Seoul	75	44.4	94	55.6	
Gyeongnam	162	63.8	92	36.2	
Daegu	100	55.6	80	44.4	
Gwangju	45	31.3	99	68.8	
Chungnam	56	62.2	34	37.8	
Type of industry (n = 1,141)					0.431
Light	149	52.5	135	47.5	
Petrochemical	100	58.8	70	41.2	
Machine and metal	266	54.5	222	45.5	
Electrical and electronics	101	50.8	98	49.2	
Company size (no. of employees) (n = 1,137)					0.211
≦4	141	49.5	144	50.5	
5-9	173	52.7	155	47.3	
10-29	211	56.3	164	43.7	
30-49	87	58.4	62	41.6	
Handling hazardous material‡(n = 1,137)					0.065
Yes	300	57.0	226	43.0	
No	315	51.6	296	48.4	
Industrial accidents for the recent 3 years (n = 1,141)					0.037
Yes	159	59.6	108	40.4	
No	457	52.3	417	47.7	
Technical/financial support§ (n = 1,123)					<0.001
Yes	159	69.1	71	30.9	
No	442	49.5	451	50.5	

*OHS : occupational health and safety problems.

^†^p-value by *χ*^2^-test.

‡Noise, Dust, DMF, Organic chemicals(Isopropyl alcohol, Xylene, Toluene, Trichloroethylene etc.), Metals(Copper, Lead, Mercury, Aluminium, Cadmium, Chromium etc.), Acids and alkalies(Hydrogen Chloride, Nitric acid, Sulfuric acid etc.), Others (Dichlorobenzidine, Arsenic, Volatile coal tar pitch, Vinyl chloride, Asbestos etc.)

§State-funded projects in supporting the health management of small businesses, Clean Workplace Program, Projects in supporting the financing for prevention of industrial accidents etc.

### Experience in facility improvement investment for industrial accident prevention

Regarding experiences in facility improvement investment for industrial accident prevention in the past three years, 39.2% of all respondents responded “yes”. By size, the percentage increased from 29.9% for those businesses with fewer than four employees to 54.1% for those with 30–49 employees, indicating that the percentage of experience in investing increases with the size of the business. The number was higher for the businesses handling hazardous materials (46.7%) compared to those that did not (33.0%). The number was also higher for businesses who had experienced an industrial accident and employees with occupational hazards in the past three years (65.0%) compared with those without (31.3%). In addition, the percentage of investments in facilities was significantly higher for the businesses that had received technical and financial support (66.8%) and those that perceived safety and health issues to be very important compared with those who did not receive technical and financial support (32.2%) and those that did not perceive safety and health issues to be very important (see Table 3).

**Table 3:** Investment in facilities for prevention of industrial accidents

	**Yes**		**No**		**p-value**^**†**^
	**N**	**%**	**N**	**%**	
Total	445	39.2	690	60.8	
Region (n = 1,135)					0.066
Gyeonggi	123	40.6	180	59.4	
Seoul	56	33.5	111	66.5	
Gyeongnam	100	39.5	153	60.5	
Daegu	68	37.8	112	62.2	
Gwangju	51	35.7	92	64.3	
Chungnam	47	52.8	42	47.2	
Industry (n = 1,135)					0.370
Light	99	35.4	181	64.6	
Petrochemical	74	43.5	96	56.5	
Machine and metal	193	39.7	293	60.3	
Electrical and electronics	79	39.7	120	60.3	
Size(person) (n = 1,131)					<0.001
≦4	85	29.9	199	70.1	
5-9	124	37.6	206	62.4	
10-29	156	42.3	213	57.7	
30-49	80	54.1	68	45.9	
Handling hazardous material (n = 1,131)					<0.001
Yes	245	46.7	200	53.3	
No	280	33.0	406	67.0	
Industrial accidents for the recent 3 years (n = 1,134)					<0.001
Yes	173	65.0	93	35.0	
No	272	31.3	596	68.7	
Technical/financial support (n = 1,116)					<0.001
Yes	153	66.8	286	33.2	
No	76	32.2	601	67.8	
Perception of OHS^*^ (n = 1,132)					<0.001
Very important	281	46.0	330	54.0	
Others	163	31.3	358	68.7	

*OHS : occupational health and safety problems.

^†^p-value by *χ*^2^-test.

### Receipt of technical and financial support from the ministry of labor and employment and the safety and health agency

The percentage of businesses that have received technical or facility improvement fund support from a government organization (Ministry of Labor and Employment and Safety and Health Agency) in the past three years was 20.5% of all respondents. By region, Gyeonggi and Chungnam exceeded the average, while machine and metal (25.3%) showed a large difference from other industries. The percentage of businesses benefiting from technical and financial support increased with their increasing size. Similarly, the percentage of businesses that responded “yes” to receiving support increased for those handling hazardous materials and those with industrial accident experience. Additionally, the percentage of businesses responding “yes” to receiving support was approximately twice as high for those perceiving safety and health issues to be very important compared to those who did not. The difference was statistically significant (see Table 4).

**Table 4:** Technical or financial support of government (Ministry of Employment and Labor, KOSHA)

	**Yes**		**No**		**p-value**^**†**^
	**N**	**%**	**N**	**%**	
Total	231	20.5	894	79.5	
Region (n = 1,125)					<0.001
Gyeonggi	89	30.1	207	69.9	
Seoul	21	12.5	147	87.5	
Gyeongnam	56	22.3	195	77.7	
Daegu	26	14.8	150	85.2	
Gwangju	17	11.8	127	88.2	
Chungnam	22	24.4	68	75.6	
Industry (n = 1,125)					0.008
Light	46	16.5	232	83.5	
Petrochemical	30	17.9	138	82.1	
Machine and metal	122	25.3	361	74.7	
Electrical and electronics	33	16.8	163	83.2	
Size (person) (n = 1,121)					0.002
≦4	42	14.9	239	85.1	
5-9	64	19.8	259	80.2	
10-29	80	21.6	290	78.4	
30-49	45	30.6	102	69.4	
Handling hazardous material (n = 1,121)					<0.001
Yes	155	29.7	367	70.3	
No	76	12.7	523	87.3	
Industrial accidents for the recent 3 years (n = 1,125)					<0.001
Yes	98	36.8	168	63.2	
No	133	15.5	726	84.5	
Perception of OHS^*^ (n = 1,123)					<0.001
Very important	159	26.5	442	73.5	
Others	71	13.6	451	86.4	

*OHS: occupational health and safety problems.

^†^p-value by *χ*^2^-test.

### Variables that affect facility investment for industrial accident prevention

In order to identify many characteristics that affect facility investment for industrial accident prevention, simple and multiple logistic regression analyses were carried out. As a result, the businesses that had an industrial accident and employees with occupational hazard in the past three years showed higher levels of facility investment for industrial accident prevention with a statistical difference with an odds ratio of 4.08 (95% CI 3.05–5.45) for the simple logistic regression analysis and 3.40 (95% CI 2.45–4.71) for the multiple logistic regression analysis. The odds ratio increased with statistical significance for the businesses that received technical and financial support compared with those that did not, as well as for the businesses perceiving safety and health issues to be very important and those handling hazardous materials compared with those that did not. Statistical significance was not found for regional and industrial differences. While the odds ratio increased with the increasing size of the businesses, the number was not statistically significant in the multiple logistic regression analysis, which corrected for related factors (Table 5).

**Table 5:** Odds ratio and 95% confidence intervals of the investment in facilities for prevention of industrial accidents by simple and multiple logistic regression

		**Unadjusted OR (95% CI)**	Adjusted OR^*^(95% CI)
Region	Gyeonggi	1.00	1.00
	Seoul	0.73 (0.49, 1.09)	0.55 (0.32, 0.93)
	Gyeongnam	0.95 (0.68, 1.34)	0.71 (0.40, 1.23)
	Daegu	0.88 (0.60, 1.29)	0.52 (0.30, 0.83)
	Gwangju	0.81 (0.53, 1.22)	0.73 (0.41, 1.27)
	Chungnam	1.63 (1.01, 2.63)	0.59 (0.32, 1.07)
Industry	Light	1.00	1.00
	Petrochemical	1.41 (0.95, 2.08)	0.75 (0.50, 1.15)
	Machine and metal	1.20 (0.89, 1.63)	1.05 (0.65, 1.69)
	Electrical and electronics	1.20 (0.83, 1.75)	0.79 (0.53, 1.17)
Size(person)	≤4	1.00	1.00
	5-9	1.41 (1.01, 1.98)	1.27 (0.87, 1.83)
	10-29	1.72 (1.24, 2.38)	1.15 (0.80, 1.67)
	30-49	2.75 (1.83, 4.16)	1.51 (0.94, 2.43)
Handling hazardous material	No	1.00	1.00
	Yes	1.77 (1.39, 2.26)	1.31 (0.97, 1.76)
Perception of OHS	Others	1.00	1.00
	Very important	1.87 (1.46, 2.38)	1.62 (1.23, 2.13)
Industrial accident for the recent 3 years	No	1.00	1.00
	Yes	4.08 (3.05, 5.45)	3.40 (2.45, 4.71)
Technical/financial support	No	1.00	1.00
	Yes	4.23 (3.11, 5.76)	3.02 (2.16, 4.23)

*Adjusted for region, type of industry, company size, shift work, hazardous material handling, perception of OHS, technical/financial support, industrial accident for the recent 3 years.

## Discussion and conclusions

Small businesses with fewer than 50 employees are economically weak with poor management conditions and are less prepared with minimal assets and ability for working environment improvements [17]. Additionally, small business owners are less able to manage burdens, and have poor working conditions, which makes it difficult for them to receive efficient industrial health services that is also low in quality [18].

Due to these characteristics, small businesses have higher industrial accident rates and poorer working conditions compared to conglomerates. The total industrial accident cases have somewhat decreased overall domestically, but the fact that the ratio for small businesses has increased to 80% supports the finding regarding accidents and working conditions [5].

As a result of surveying the importance of safety and health issues in small businesses with fewer than 50 employees, the number of businesses that perceive safety and health issues to be very important accounted for 54.1%, constituting the majority. This is seemingly different from the finding of a previous study in which 29.6% chose “very interested” for the question regarding the level of interest by business owners for workplace health management [19]. However, this difference with the results of this study was not big when the response “somewhat interested” was included in the results of the previous study, although the intent was somewhat different in the previous studies as it asked for interest levels. As a result, in contrast to the existing perception that there is a low interest in health and safety of small businesses, a high interest was found with a high level of awareness for the importance of these issues. This can be interpreted as reflecting a trend regarding expanding demands on safety and health in domestic businesses [13].

However, result of the multiple logistic regression analysis related to the investment in preventing industrial accidents showed that the influential factors were safety and health issue awareness in facility investment experiences, industrial accident history, and receiving technical and financial support. Businesses that answered safety and health issues as “very important” showed that facility investment experience was 1.6 times that of the businesses that did not respond similarly. Such differences can be interpreted as practical facility investment achieved by going beyond simply perceiving safety and health issues as important and prioritizing safety and health issues from a business management perspective. For this, the awareness of safety and health issue importance should be allowed to lead into actual safety and health behaviors, while business owners as well as employees actively demand and participate in safety and health.

Therefore, receiving technical and financial support or past industrial accidents were important factors for increasing safety and health issue awareness. This is evident from the survey regarding the factors that determined facility investment for industrial accident prevention. This showed that the businesses that considered safety and health issues to be very important showed a higher ratio compared to those that did not.

It is difficult for small businesses to independently invest in industrial accident prevention and they rely on related technical and financial support. In the survey regarding receiving technical and financial support, businesses that did showed a 20% higher response for “very important” compared to those that did not receive support. Upon examining previous studies, there were various assessments regarding health technology support projects for small businesses, which showed conflicting assessments that the projects were essential for small businesses along with the assessment that they did not sufficiently reflect the opinions of the businesses and service providers [10]. However, according to a study’s result, despite many negative assessments, the majority of business owners responded that it was desirable to continue the projects, positively assessing the technical and financial support projects [20]. Taking these findings together, it can be interpreted that technical and financial support for a business is closely related to the business’ awareness of safety and health issue importance.

In the survey that asked whether they have received technical and financial support from the Ministry of Employment and Labor, and the Safety and Health Agency, 20.5% gave an affirmative answer, reflecting that only a minority of businesses were benefiting from this support. Though most small businesses are aware of the importance safety and health issues, it can be said that the support for realistically implementing this is still inadequate. Furthermore, in the case of businesses with fewer than five employees, the percentage of businesses benefiting from this support was 14.9%, which was far below the percentage for those with 30–49 employees (30.6%). Businesses with fewer than five employees are vulnerable in terms of safety and health management, with particularly low awareness for dangers, while it is impossible to realistically divide work related to safety due to the small number of employees [21]. Therefore, it is necessary to expand government support in addition to providing more benefits for small businesses with fewer than five employees when selecting businesses in the future.

According to the current Industrial Safety and Health Act, small businesses with fewer than 50 employees are not obligated to perform health management or appoint a health manager [22]. Furthermore, projects that aim to supplement issues and provide technical and financial support for small businesses are currently being carried out. These projects include the following: “Safety and Health Keeper Project”, [23] which performs essential safety and health management work for small businesses; the “Clean Business Development Project”, [24] which supports improvement regarding hazardous materials or risk factors; and the health management support fiscal agent business for small businesses. There are other various forms of support projects as well.

However, thus far, these show many limitations as fundamental measures for industrial accident prevention. In the case of the “Clean Business Development Project”, the workplace injury rate in the 2009 survey for businesses designated as clean businesses and those not designated as clean businesses were 1.69% and 1.43%, respectively. There was only a 0.26% difference. In addition, according to the 2010 data, the percentage of businesses previously certified as clean businesses that applied for re-certification was only 8.2%, with only approximately half (50.3%) being re-certified [25]. This shows that while technical and financial support projects are being undertaken, there are many problems regarding maintenance and follow-up.

Regarding the “Clean Business Development Project”, the businesses requiring re-certification must pay for the cost of facility improvements themselves with no incentives provided for receiving re-certification. Therefore, for small businesses with fewer than 50 employees that are financially weak, the ratio of re-certification applications is low despite high awareness of safety and health issue. Accordingly, as a measure for this, small business policies need to be more accessible, while thorough follow up management, such as inducing facility improvement through a continuous government technical and financial support, is necessary for the businesses that are doing this. Such facility improvement through investment is believed to be very important in industrial accident prevention.

Therefore, regular business education is necessary to improve awareness of safety and health issue importance while various onsite participation incentives should be provided, so that these efforts can lead to actual investments. Currently, Korea shows great improvement in the introduction and operation of such systems, but there are areas that are neglected due to the lack of awareness regarding their necessity. Therefore, it is necessary to thoroughly modify existing operating systems in order to achieve perfect maintenance and follow up management.

The strengths of this study are that it identifies safety and health issue awareness according to each variable as well as the facility investment experiences by surveying safety and health issue awareness and its status in small businesses. This included the occurrence of industrial accidents within the past three years, the safety and health issue awareness, and technical and financial support. It also surveyed basic business information, such as business size, industry, and handling of hazardous materials through detailed questionnaires. In particular, the study analyzed the safety and health issue awareness and facility investment experiences as well as the industrial accident occurrence in the past three years to estimate the causal relationships between them. It showed which cases resulted in industrial accident preventive activities for small businesses. In addition, representativeness was ensured by using a sample extraction method through a population analysis and the representativeness was sufficient for the sample as the study was conducted with sample businesses selected through an objective and systematic selection method. Furthermore, as it included the small businesses in the six regional industrial complexes nationwide, it satisfied the conditions for representativeness and is more representative compared to existing studies. Thus, the study is significantly provides the fundamental data for industrial accident prevention activities in small businesses with fewer than 50 employees.

However, this study’s limitation was that it did not sufficiently reflect employee views as the study targeted the business owners or the managerial-level employees, even though each business had a representative for each region. Additionally, there is a possibility of information bias as safety and health issue awareness, facility investment experiences, and industrial accident experiences were obtained via surveys rather than through objective data collection. For the businesses with industrial accidents, the possibility of over-exaggerating technical and financial support as well as facility investment experience cannot be eliminated while, for the businesses with low safety and health awareness, it is possible that they answered lower than the reality regarding their investment experience. Therefore, additional research is necessary to determine the effects on facility investment in relation to safety and health.

In addition, as it was a cross-sectional study, another limitation was in its limited ability to explain the causal relationship even though a relationship may exist between safety and health awareness and facility investment experience.

As a result of this study, it was found that awareness regarding safety and health issues was high if small businesses had experienced industrial accidents or received technical and financial support. Additionally, more investment was made for industrial accident prevention if the safety and health issues were perceived to be important or if the business had previously had an experience with industrial accidents or received technical and financial support. Therefore, the ultimate goal of small business safety and health projects is in recognizing their importance and prioritizing safety and health issues in terms of business management. For this, various support projects are currently being undertaken with limited businesses, and inadequate maintenance and follow up management. Therefore, regular business education is necessary for the improvement of safety and health issue awareness and it is necessary to provide various onsite participation incentives, so the efforts lead to actual investment, while thoroughly modifying existing operating systems to achieve optimum maintenance and follow up management.

## Abbreviations

OHS: Occupational health and safety problems.

## Competing interests

The authors declare that they have no competing interests.

## Authors’ contributions

JS Park, JT Park, DS Kim and HJ Kim conceived and designed the study. JS Park was involved in writing the manuscript. All authors developed research model, especially JS Kim collected data and JS Park, HR Jeong and SJ Hong analyzed the statistics and wrote the manuscript. All authors read and approved the final manuscript.
